# Clozapine Suppresses the Gene Expression and the Production of Cytokines and Up-Regulates Cyclooxygenase 2 mRNA in Human Astroglial Cells

**DOI:** 10.3390/brainsci12121703

**Published:** 2022-12-12

**Authors:** Yael Yuhas, Shai Ashkenazi, Eva Berent, Abraham Weizman

**Affiliations:** 1 Laboratory of Pediatric Infectious Diseases, Felsenstein Medical Research Center, Sackler Faculty of Medicine, Tel Aviv University, Petach Tikva 4941492, Israel; 2 Laboratory of Molecular and Biological Psychiatry, Felsenstein Medical Research Center, Petach Tikva 4941492, Israel; 3 Department of Psychiatry, Sackler Faculty of Medicine, Tel Aviv University, Tel Aviv 6997801, Israel; 4 Research Unit, Geha Mental Health Center, Petach Tikva 4941492, Israel

**Keywords:** schizophrenia, neuroinflammation, clozapine, immune system, proinflammatory cytokines, TNFα, IL-1β, IL-8, human astroglial cells, COX2

## Abstract

Schizophrenia (SCZ) is a chronic neurodevelopmental psychotic disorder. The immune system and neuroinflammation seem to play a central role in the pathophysiology of SCZ. Clozapine is an effective atypical antipsychotic used for treatment-resistant SCZ. Life-threatening side effects, such as myocarditis, limit its use. We investigated the immunomodulatory effects of clozapine in an astroglial model of neuroinflammation. We thus assessed the effect of clozapine on the production of inflammatory mediators in human-derived astroglial (A172) cells, stimulated with a cytokine mix (TNFα, IL-1β, IFNγ). RT-PCR and ELISA analyses demonstrated that clozapine suppressed gene expression and production of TNFα, IL-1β and IL-8 and increased COX2 mRNA 24 h after stimulation. Clozapine inhibited Akt phosphorylation induced by the cytokine mix at 10 min and 40 min, as assessed by Western blot analysis with anti-pT308Akt antibody. Pretreatment with the Akt inhibitor MK-2206 increased COX2 gene expression in cytokine-stimulated cells, suggesting that Akt inhibition may be involved in COX2 gene expression upregulation. Clozapine may possess dual beneficial effects: inhibiting astroglial production of proinflammatory cytokines, thus attenuating neuroinflammation, and upregulating COX2 expression that may be relevant to improvement of neural functioning while accounting for some of its detrimental effects. Patients with TRS and neuroinflammatory markers may benefit particularly from clozapine treatment.

## 1. Introduction

Schizophrenia (SCZ) is a chronic devastating neurodevelopmental psychotic disorder that affects about 1% of the population. The first line of treatment is the use of typical and atypical antipsychotics, but when this approach fails, clozapine is the last resort for treatment-resistant schizophrenia (TRS) [1]. It was suggested that failure of the first typical or atypical antipsychotic already justifies switching to clozapine [2] There is strong evidence that the immune system and neuroinflammation play a central role in the pathophysiology of SCZ [3,4,5,6,7,8,9]. Epidemiological studies have demonstrated that various infections and autoimmune disorders in the prenatal period or in early childhood, as well as chronic stress and genetic susceptibility, are associated with increased risk of developing SCZ later in life. It thus seems that over-activation of the inflammatory response may be an underlying mechanism [6,10]. Recent studies have shown a linkage between SCZ-associated chromosomal loci and inflammatory markers such as pro-inflammatory cytokine gene polymorphisms, major histocompatibility complex (MHC) and Toll-like receptors (TLRs) [4,11,12,13]. Neurodevelopmental dysfunctions involving neuroinflammation potentiated by kynurenine metabolites may be responsible for altered dynamic functional brain connectivity and cognitive dysfunctions in SCZ [14,15,16].

Glial cell types in the brain include microglia, oligodendrocytes and astrocytes. It was suggested that pro-inflammatory cytokines released by over-active microglia affect the function of the dopaminergic and glutamatergic systems and the cortical integrity and connectivity, relevant for cognitive deficits in schizophrenia [1,17,18,19,20]. Astrocytes in particular play a major role in the synaptic metabolism of glutamate and monoamines, synaptic pruning, coordination of immune responses, and modulation of metabolic exchange through the blood–brain barrier [18]. Thus, astrocyte over-activity may be related to the synaptic transmitter and neuroinflammatory theories of schizophrenia, which may be relevant to the etiopathology of schizophrenia and the cognitive deficits associated with the disorder [20]. Translational experimental research in glial cell models has been shown to be valid for the study of human diseases such as SCZ [21].

Numerous studies have investigated blood cytokine levels in patients with SCZ, reporting inconsistent findings [5,22]. In a meta-analysis of 40 studies, Miller et al. found that patients with a first episode psychosis or acute relapse had significantly elevated levels of the pro-inflammatory cytokines interleukin (IL)-1β, IL-6, tumor necrosis factor (TNF)α, interferon (IFN)γ and IL-12. In SCZ patients treated with antipsychotics, a significant decrease was noted in IL-6, IL-1β and IFNγ cytokines and a rise in IL-12 and IL-2 receptors [23]. In another meta-analysis of 18 studies, Goldsmith et al. reported that in chronically ill patients with SCZ the levels of IL-6, IL-1β and soluble IL-2 receptor (sIL-2r) were significantly higher compared to controls. In patients with acute SCZ the levels of IL-6, TNFα, sIL-2r, and IL-1 receptor antagonist (IL-1ra) were increased. Similar patterns of cytokine alterations were found in bipolar and major depressive disorders [24].

Inflammatory processes are not restricted to the peripheral immune system, as aberrant cytokine levels were also found in the central nervous system (CNS). Two recently published meta-analyses found significantly higher levels of IL-1β, IL-6 and IL-8 in the cerebro-spinal fluid (CSF) of SCZ patients compared to healthy controls [25,26]. Neuroinflammatory manifestations, such as an activation of microglial cells associated with loss of brain volume, were demonstrated in SCZ [3,7,11,12].

Strong evidence on the involvement of inflammation in SCZ points to the possible beneficial effects of anti-inflammatory agents in SCZ. Indeed, various antipsychotic and antidepressant medications possess immunomodulatory properties [3,27,28,29,30].

Clozapine, a tricyclic dibenzodiazepine, is an effective atypical antipsychotic agent used for TRS. However, some life-threatening side effects, such as myocarditis, cardiomyopathy, agranulocytosis, and seizures, limit its use [31,32,33]. Several studies have investigated the effects of clozapine on inflammatory mediators in blood cells in vitro and in patients with SCZ. The results were inconsistent, showing pro- and anti-inflammatory effects for the same cytokine, depending on the cell type and the inducer of the inflammatory response [34]. Notably, clozapine reversed protein changes induced in astrocytes by dizocilpine (MK-801), a non-competitive NMDA receptor antagonist that is used in animal models to provoke schizophrenia-like positive and negative symptoms [35].

Only a few studies were published regarding the effects of clozapine on the production of inflammatory mediators within the brain. The main sources of proinflammatory mediators in the CNS are microglial and astroglial cells, which resemble peripheral macrophages. In a model of primary neuron-glia cultures and in a rat microglial cell line Hu et al. [36] showed clozapine attenuated lipopolysaccharide (LPS)-induced microglial activation by reducing the generation of reactive oxygen species (ROS), nitric oxide (NO) and TNFα, thus protecting dopaminergic cells from degeneration. It has also been reported that in primary rat microglial cells, clozapine suppresses LPS-induced nuclear factor kappa B (NF-kB) activity, gene expression of IL-1β, IL-6, inducible nitric oxide synthase (iNOS) and cyclooxygenase (COX)2, as well as decreasing the production of NO [37]. The same was reported for the mouse BV2 microglial cell line. In a recent study, performed in rat primary microglial cells, stimulated by polyinosinic: polycytidylic acid (Poly I:C), Giridharan et al. [38] showed that clozapine inhibited the release of IL-1α, IL-1β, IL-2 and IL-17 and reduced the level of Poly I:C activated NACHT, LRR, and PYD domains-containing protein 3 (NLRP3) inflammasome expression. 

The above-mentioned studies were conducted in rodent microglial cells stimulated with LPS or Poly I:C (PIC) mimicking Gram-negative bacterial or viral infection, respectively. It is unclear whether the changes that occur in response to clozapine in rodent glial cells, in a model of infection, represent the effects of clozapine in human glial cells. The present study investigated the in vitro effect of clozapine on inflammatory processes in human-derived astroglial cells. There is strong evidence that astrocytes play an essential role in neuroinflammation and in the pathophysiology of SCZ [18,19,20,39]. This led to choosing the current human astroglial cellular model for the evaluation of the immunomodulatory activity of clozapine. This activity was examined both in unstimulated cells and in cells stimulated with a cytokine mix, TNFα, IL1-β, IFNγ, as these cytokines are elevated during both infective and sterile inflammation. We investigated the effect of clozapine on TNFα, IL1-β, IL-8 and cyclooxygenase 2 (COX2) as they are major mediators in inflammation [40,41,42]. 

## 2. Materials and Methods

### 2.1. Reagents

The cell culture medium and the supplements were obtained from Biological Industries (Beit HaEmek, Israel). The recombinant human IL-1β, IFNγ, and TNFα were provided by ProSpec-Tany TechnoGene Ltd. (Rehovot, Israel).

Clozapine was purchased from Sigma Chemical (St. Louis, MO, USA) and MK-2206 from Sigma-Aldrich (St. Louis, MO, USA). Stock solutions of clozapine (10 mM) and MK-2206 (29 mM) were prepared using dimethyl sulfoxide (DMSO; Sigma, St. Louis, MO, USA) and diluted in culture medium to final concentration of 10 µM and 1 µM, respectively. DMSO concentration in the cell cultures was ≤0.1% in all experiments. 

### 2.2. Cell Culture

American Type Culture Collection (ATCC, Manassas, VA, USA) was the source of the human-derived glioblastoma (astroglial) A172 cells. The cells were maintained at 37 °C in Dulbecco modified Eagle medium (DMEM) and in 5% CO_2_ atmosphere in a humidified incubator. The medium was supplemented with 10% heat-inactivated fetal bovine serum and L-glutamine (2 mM) and with penicillin (100 U/mL), streptomycin (100 mg/mL), and nystatin (12.5 U/mL).

The cells were incubated in serum-free medium for 24 h and subsequently exposed to a cytokine mix (mix) of TNFα, IL-1β, and IFNγ (100 ng/mL each) with or without clozapine (10 µM; a concentration based on previous in vitro experiments [43], or to clozapine alone. Clozapine was added to the cell culture 45 min prior to adding cytokine mix. 

Evaluation of cells viability was performed by the neutral red uptake viability assay, as described in a previous publication [44], and yielded no differences in viability among the differently treated cells. 

### 2.3. Cytokine Array

One milliliter of culture medium was centrifuged to remove cell debris. and the supernatants were collected and analyzed according to the manufacturer’s instructions, using a Proteome Profiler Array Human Cytokine Panel A kit (R&D Systems, Minneapolis, MN, USA) as described in a previous publication [45].

### 2.4. Real-Time Polymerase Chain Reaction (RT-PCR)

Trizol Reagent kit (Invitrogen, Life Technologies, Carlsbad, CA, USA) was used to isolate total RNA from A172 cells, according to the manufacturer’s instructions. The RNA concentrations were evaluated using a NanoDrop One (Thermo Scientific Pierce, Waltham, MA, USA). The RNA was transcribed to cDNA using the High-Capacity cDNA RT (Reverse Transcriptase) kit with random primers (Applied Biosystems, Foster City, CA, USA). Real-time PCR was performed using TaqMan Assay-on-Demand and specific TaqMan Gene Expression were used to evaluate the mRNA levels of TNFα, IL-1β, IL-8 and COX2 in the cDNA samples (Applied Biosystem, ID assay Hs00174097_g1 for TNFα, ID assay Hs00174097_m1 for IL1-β, ID Hs00174103_m1 for IL-8, ID COX2/PTGS2:Hs 001153133_m1 for COX2). The transcript levels for all cytokines and COX2 were normalized to human RPLPO ribosomal protein transcript level. RT-PCR reactions were conducted in a Step One Plus RT-PCR system (Applied Biosystems). All reactions were performed in triplicates. The gene expression levels were calculated by relative quantification using the ddcT methods as described in previous literature [46]. 

### 2.5. Cytokine Determination

Cell cultures supernatants were harvested and centrifuge to remove cells debris and assessed for concentrations of TNFα, IL-1β and IL-8 according to the manufacturer’s instructions by an enzyme-linked immunoassay (ELISA) kit from R&D Systems (Minneapolis, MN, USA). 

### 2.6. Western Blot

A172 cells were seeded in 3 cm dishes (0.5 × 10^6^ cells/plate), grown for 24 h, and then incubated in serum- and antibiotic-free medium for another 24 h This was followed by exposure to clozapine (10 µM) and cytokine mix. At the indicated times, the cells were washed twice with cold phosphate-buffered saline, lysed with a buffer (50 mM Tris, pH 6.8, 2% sodium dodecyl sulfate, 10% glycerol), and denatured at 95°. Equal amounts of protein (40 μg) from total cell extracts, estimated by use of a bicinchoninic acid reagent (Pierce Biotechnology, Rockford, IL, USA), were loaded onto a 10% sodium dodecyl sulfate-polyacrylamide gel and transferred to a polyvinylidene difluoride membrane (Amersham Biosciences, Piscataway, NJ, USA). Nonspecific binding sites were blocked by 5% milk in TBST (20 mM Tris, pH 7.8, 150 mM NaCl, 0.1% Tween 20) at room temperature. The membranes were then incubated overnight at 4 °C with either rabbit anti-phospho-Akt (Thr308) antibodies, or mouse anti-Akt monoclonal antibodies (Cell Signaling Technology, St. Louis, MO, USA) or goat anti-actin antibodies (Santa Cruz biotechnology, Santa Cruz, CA, USA). Subsequently, the membranes were washed and further incubated with corresponding anti-mouse (IRDye 680 conjugated) or anti-rabbit (IRDye 800 conjugated) secondary antibodies. Odyssey Infrared Imaging System (LI-COR Biosciences, Lincoln, NE, USA) was used to visualize the signals and quantify them with the corresponding Odyssey software.

### 2.7. Statistical Analysis

Statistical analysis was conducted with SPSS v. 24.0 (IBM Co., Armonk, NY, USA). An unpaired *t*-test was used to compare results between treatments. Results are presented as mean± SEM. Significance was set at *p* < 0.05. 

## 3. Results

### 3.1. Effects of Clozapine on Inflammatory Mediators: Cytokine Array Analysis

To determine the effect of clozapine on inflammatory processes, we used a cytokine array assay screening of 35 inflammatory mediators. Cells were incubated with medium alone (non-inflammatory condition), clozapine (10 µM), cytokine mix (TNFα, IL-1β, IFNγ; inflammatory condition) or cytokine mix in the presence of clozapine (10 µM). The supernatants were collected after 24 h and assayed for cytokine levels. 

#### 3.1.1. Non-Inflammatory Condition

**Figure 1:**
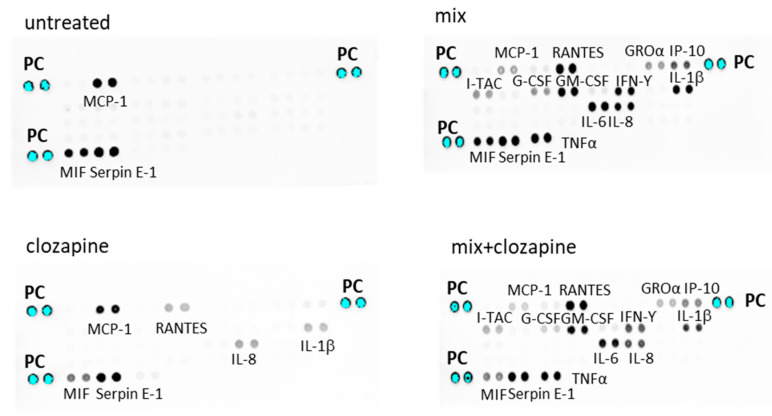
Effects of clozapine on the protein production of inflammatory mediators. A172 cells were treated with either clozapine (10 µM) alone, or the cytokine mix, or the cytokine mix with clozapine. The culture medium was collected 24 h after stimulation and analyzed using a human cytokine array. PC—positive control. The figure shows a representative blot of one of two similar experiments.

The untreated A172 cells spontaneously secreted monocyte chemotactic protein 1 (MCP1), Serpine E-1 and migration inhibitory factor (MIF). Treatment with clozapine alone induced modest secretion of IL-1β, IL-8 and regulated upon activation, normal T cell expressed and presumably secreted (RANTES) also known as chemokine C- motif ligand 5 (CCL5), which were undetectable in untreated cells. Additionally, clozapine reduced the spontaneous secretion of MIF and MCP1 (Figure 1).

#### 3.1.2. Inflammatory Condition

Incubation of the A172 cells with cytokine mix, stimulated the production of the following mediators: RANTES, growth-regulated oncogene alpha (GROα aka CXCL-11), IP-10 (CXCL10), granulocyte colony-stimulating factor G-CSF, granulocyte-macrophage colony-stimulating factor (GM-CSF), IFN-inducible T-cell chemoattractant (I-TAC), IL-6 and IL-8. 

The addition of clozapine (10 µM) to the cytokine mix reduced the secretion of GROα, IP-10, G-CSF, I-TAC, IL-1β and IL-8. A small reduction was also observed in TNFα, IFNγ and IL-6. Lower levels of MCP-1 and MIF, observed in cells treated with clozapine alone, were also observed when clozapine was added to the cytokine mix (Figure 1, right panel).

### 3.2. Inhibitory Effect of Clozapine on TNFα, IL-1β, and IL-8 Gene Expression and Protein Production

Due to the major role of TNFα, IL-1β and IL-8 in inflammation, we investigated the effect of clozapine on the production of these cytokines, 24 h after stimulation with cytokine mix, using mRNA RT-PCR analysis and ELISA assay. 

**Figure 2:**
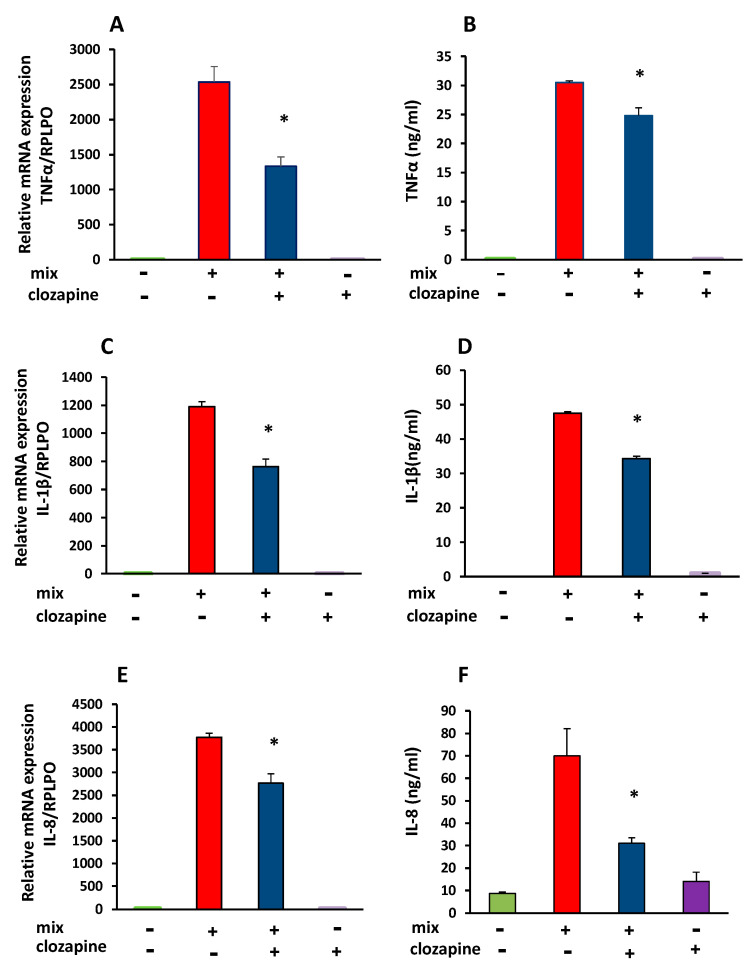
Effects of clozapine on TNFα, IL-1β, and IL-8 gene expression and protein production. Data are presented as mean ± SEM. (**A**,**C**,**E**): RT-PCR analyses of mRNA expression in A172 cells treated with cytokine mix (mix) and clozapine (10 µM) for 24 h. (**A**) TNFα (*p* < 0.001, mix vs. mix with clozapine *n* = 4 in each group). Data represent the results of one of 5 similar experiments. (**C**) IL-1β, (*p* = 0.003 mix vs. mix with clozapine, *n* = 4 in each group). Data represent the results of one of 3 similar experiments. (**E**) IL-8, (*p* = 0.004, mix vs. mix with clozapine *n* = 4 in each group). Data represent the results of one of 4 similar experiments. (**B**,**D**,**F**): TNFα, IL-1β, and IL-8 levels in cell supernatants 24 h after stimulation with cytokine mix in the presence or absence of clozapine (10 µM). (**B**) TNFα, (*p* = 0.007, *n* = 3 in each group). (**D**) IL-1β (*p* < 0.001, *n* = 3 in each group). (**F**) IL-8 (*p* = 0.02, *n* = 4 in each group). Details on all experiments are presented in the text. * *p* < 0.01 vs. mix.

Clozapine (10 µM) reduced the TNFα mRNA by 36.6 ± 6.5% (mean of 5 separate experiments, *n* = 4 in each group; t = 5.68, df = 8, *p* < 0.001) in the cytokine mix-stimulated cells. Clozapine also attenuated TNFα protein production, 24 h after incubation with cytokine mix. Concentration of TNFα in the supernatant was reduced by 19 ± 4% compared to cells incubated with cytokine mix alone (*n* = 3 in each group, t = 4.07, df = 4, *p* = 0.015). Clozapine did not affect TNFα mRNA or TNFα release in unstimulated cells, namely cells treated with clozapine alone without the addition of cytokine mix (Figure 2A,B).

Incubation of the cytokine-stimulated cells with clozapine also reduced IL-1β mRNA by 36.4 ± 5.2%, (mean of 3 experiments, *n* = 4 in each group; t = 4.78, df = 6 *p* < 0.001), as well as that of IL-1β secretion, which was reduced by 27.7 ± 1.4% (t = 8.48, df = 4, *p* < 0.001, *n* = 3 in each group). Similar to the findings in the cytokine array assay, a non-significant increase in IL-1β concentration was detected in the supernatants after incubation with clozapine alone (Figure 2C,D).

Clozapine also significantly reduced cytokine-stimulated IL-8 mRNA expression by 23.9 ± 3.6% (mean of 4 separate experiments, *n* = 4 in each group; t = 13.46, df = 6, *p* < 0.0001), and IL-8 protein level in supernatants by 65 ± 4.1% (t = 8.51, df = 6, *p* < 0.001, *n* = 4 in each group) (Figure 2E,F).

### 3.3. Clozapine Up-Regulates Cyclooxygenase 2 mRNA Expression

**Figure 3:**
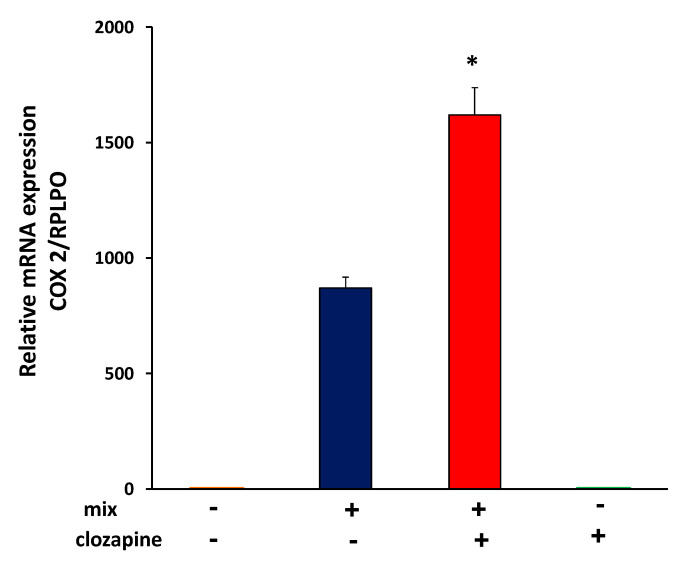
Effects of clozapine on COX2 gene expression. A172 cells were treated with clozapine (10 µM) 45 min prior to the addition of cytokine mix, left for a duration of 24 h. COX2 mRNA levels were determined by RT-PCR (*p* < 0.01, *n* = 4 in each group). Data are expressed as Mean ± SEM and represents one of 5 similar experiments. Details on all 5 experiments are presented in the text. * *p* < 0.01 vs. mix.

COX2, the inducible isoform of COX, which initiates the formation of prostaglandins from arachidonic acid, is strongly associated with inflammation [40,47]. It has been reported that expression of genes involved in prostaglandin E_2_ (PGE2) production differs in older (above 40 years of age) patients with SCZ, compared to younger patients with SCZ or healthy controls of similar age [48]. We thus investigated the effect of clozapine on COX2 expression. Incubation of the cells with cytokine mix induced COX2 mRNA elevation, and the addition of clozapine to the cytokine mix for 24 h increased the effect by 86 ± 7.5% (average of 5 separate experiments, *n* = 4 in each group; t = 11.67, df = 8, *p* < 0.0001) (Figure 3). 

### 3.4. Clozapine Inhibits Akt Phosphorylation

It has been reported that clozapine regulates Akt activation in various cellular models. However, results were inconsistent, with some studies reporting that clozapine inhibits Akt phosphorylation [37,49,50,51] while others demonstrating an increase [52,53,54]. 

**Figure 4:**
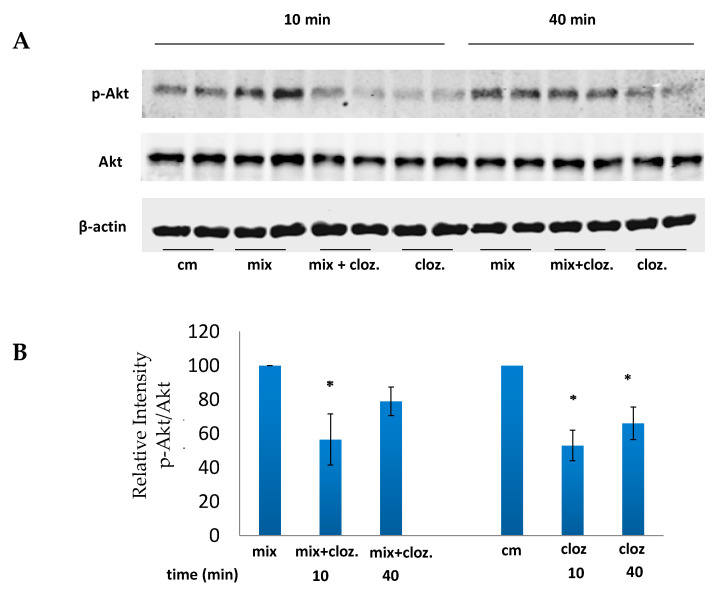
Effects of clozapine on Akt phosphorylation. (**A**) A172 cells were incubated with cytokine mix, or cytokine mix plus clozapine 10 µM or clozapine alone. Cytoplasmic extracts were prepared at the indicated times and subjected to Western immunoblotting with antibodies for the phosphorylated form of Akt (p-Akt-Thr-308). p-Akt = phosphorylated Akt; cm = control medium (i.e., medium alone); The blot is representative of one of three experiments with similar results. (**B**) Densitometric analysis of 3 independent experiments presented as relative intensity. The results are presented as mean ± SEM of 3 experiments (t= 2.84, df = 6, *p* < 0.05 for mix vs. mix +clozapine after 10 min; t = 5.06, df = 6, *p* < 0.01 for cm vs. clozapine after 10 min; t = 3.5, df = 4, *p* < 0.05 for cm vs. clozapine after 40 min). * *p* < 0.05 vs. mix or cm.

We investigated the effect of clozapine on Akt phosphorylation in A172 cells stimulated with cytokine mix. As shown in Figure 4, incubation of A172 cytokine-stimulated cells with clozapine reduced Akt phosphorylation within 10 min. A small decrease in Akt phosphorylation persisted at 40 min, and it became undetectable after 6 h (data not shown).

**Figure 5:**
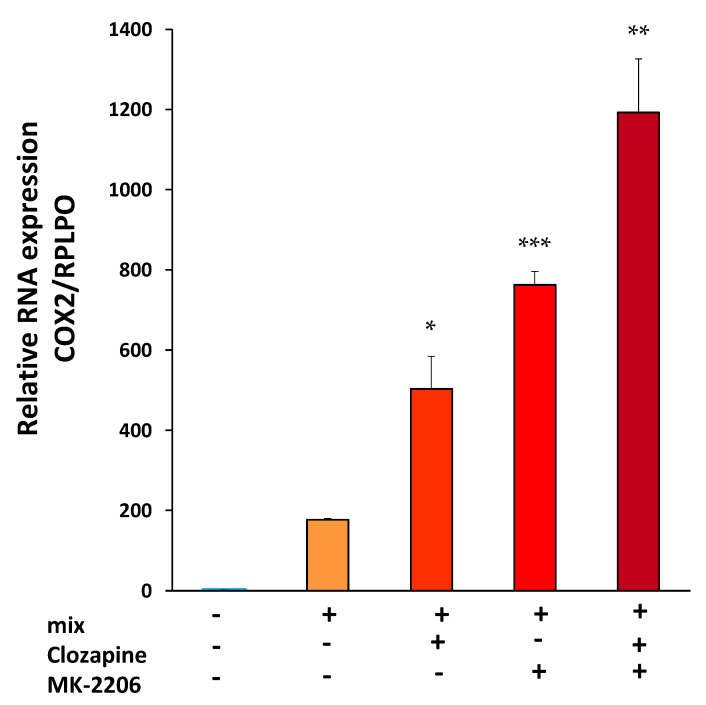
Effects of clozapine and MK2206 on COX2 gene expression. A172 cells were treated with clozapine (10 µM) alone, MK2206 (1 µM) alone, and a combination of both, 45 min before the addition of cytokine mix (mix). COX2 mRNA levels were determined by RT-PCR. (t = 3.98, df = 6, *p* < 0.01 for mix with clozapine vs. mix, t = 17.20, df = 6, *p* < 0.0001 for mix with MK vs. mix, t = 4.40, df = 6, *p* < 0.005 for mix with both clozapine and MK-2206 vs. mix with clozapine; *n* = 4 in each group). The figure represents one of two experiments with similar results. Data are presented as mean ± SEM. * *p* < 0.01 mix with clozapine vs. mix. ** *p* < 0.005 mix with both clozapine and MK-2206 vs. mix with clozapine. *** *p* < 0.0001 mix with MK-2206 vs. mix.

To determine whether clozapine inhibition of Akt activation is involved in the upregulation of COX2 expression, we examined the effect of the Akt inhibitor MK-2206 on COX2 gene expression in cells exposed to cytokine mix and to cytokine mix in the presence of clozapine. Pretreatment with MK-2206 and clozapine, 45 min before cytokine mix stimulation, increased COX2 mRNA beyond the increase caused by cytokine mix in the presence of clozapine alone. Moreover, pretreatment with MK-2206 alone of cells stimulated with cytokine mix, increased COX2 expression significantly (Figure 5). These results indicate that Akt inhibition by clozapine may be involved in the increase in COX2 expression. MK-2206 and clozapine alone did not affect COX2 expression (data not shown).

Table 1 below presents the major results of the study.

## 4. Discussion

The present study demonstrates that clozapine modulates the gene expression and the production of pro-inflammatory mediators in cultured, human-derived, astroglial cells. In vitro anti-inflammatory effects of clozapine were previously reported in human blood cells stimulated with LPS or PIC. The results were inconclusive with various studies showing contradictory effects for the same cytokine. It was suggested that the methodology employed, especially the cell type and the inducer, play a crucial role in the immunomodulatory effects of clozapine [34].

Neuroinflammation has been implicated in the pathophysiology of SCZ and has been related to excessive release of pro-inflammatory cytokines from activated glial cells [31]. We therefore chose to examine the effect of clozapine on the production of pro-inflammatory mediators in a human astroglial cellular model, which, together with microglial cells, constitute the main source of cytokines in the CNS, and play a pivotal role in neuroinflammation [19,20]. We examined the effect of clozapine on astroglial cells, both in unstimulated condition and stimulated with cytokine mix. Previous studies, investigating the in vitro effects of clozapine in the CNS, were performed in mouse or rat microglial cells, stimulated with LPS or PIC, mimicking a Gram-negative bacterial and viral infection, respectively. 

In cytokine mix stimulated astroglial cells we found that clozapine suppressed the gene expression and the production of TNFα, IL-1β and IL-8. These results are consistent with those of Hu et al. [36] who showed that clozapine attenuates LPS-induced TNFα-release in human and rat microglial cell lines. Jeon et al. [37] reported that in primary rat microglial cells and mouse BV2 cells, stimulated with LPS, clozapine reduces IL-1β expression, which is also in line with our findings. However, contrary to our results, they found that clozapine also reduces COX2 expression, but as suggested by Baumaster et al. [34], this discrepancy may be related to differences in cellular models. 

In this context, it is of note that human and rodents differ in their ability to produce NO; while human monocytes and macrophages do not produce inducible NO, rat and mouse macrophages and monocytes are efficient producers of NO [55,56]. NO is a signaling molecule with numerous biological activities, which regulates several transcription factors including NFkB that modulates the expression of proinflammatory genes such as IL-1β, IL-6, iNOS and COX2 [57,58]. It is likely, therefore, that the diverse effects of clozapine observed in humans vs. rodents, may be related, at least partly, to the different patterns of inducible NO production.

In unstimulated astroglial cells the cytokine array assay demonstrated that clozapine increased the release of IL-1β, IL-8 and RANTES, and reduced the level of constitutively secreted MCP-1 and MIF. The increases in IL-1β and IL-8 in the unstimulated cells were also demonstrated by ELISA, but, did not reach significant values. However, no such increase was detected in mRNA expression of either IL-1β or IL-8, indicating that in unstimulated cells clozapine increases the level of these cytokines probably by a post-transcriptional regulatory mechanism. Reale et al. [5] found that, compared to healthy controls, peripheral blood mononuclear cells of patients with SCZ produce significantly higher levels of constitutively and LPS-induced MCP-1, MIP-1a, IL-8 and IL-18, and lower RANTES and IFNγ levels. In patients treated with risperidone, olanzapine or clozapine basal and LPS-induced production of RANTES and IL-18 were higher, while such production of MCP-1 was lower. This is in line with our observation that in unstimulated human astroglial cells, clozapine induced an increase in RANTES and a decrease in MCP-1.

Notably, contrary to the attenuating effect of clozapine on the production of TNFα, IL-1β and IL-8, in cytokine mix-stimulated astroglial cells, clozapine strongly upregulated COX2 gene expression. COX enzymes catalyze arachidonic acid (AA) to produce prostaglandins. There are two COX iso-enzymes: COX1, which is constitutively expressed, and COX2, which is inducible and upregulated by proinflammatory mediators and growth factors. In the CNS, unlike in other tissues, COX2 is constitutively expressed mainly in neurons. In astroglial and microglial cells, COX2 is undetectable in physiological conditions, but is strongly upregulated by exposure to proinflammatory mediators [59]. 

It has been hypothesized that inappropriate levels of membrane fatty acids may contribute to the pathophysiology of SCZ [60] and that activation of AA hydrolysis and imbalance in the levels of COX1 and COX2 may be involved in the disorder [61]. Indeed, abnormalities in membrane phospholipids have been found in SCZ and lower levels of AA have been reported in red blood cell membranes collected from patients with SCZ [62]. In a recent study Yang et al. [61] examined the mRNA levels of 19 genes, related to the phospholipase A2 (PLA2)—COX2 pathway in leukocytes of patients with SCZ. They detected significant differences in the expression of 6 genes, between individuals with SCZ and healthy controls, including upregulation of cytosolic PLA2 (cPLA2) and downregulation of COX2. Lower levels of COX2 were also demonstrated in plasma of patients with SCZ, aged 40 years and older [48]. Another study reported lower levels of cytosolic PGE2 synthase, an enzyme that generates PGE2 from prostaglandin H2, in postmortem tissue from the frontal cortex of patients with SCZ as compared to healthy controls [63].

There is a body of evidence showing that COX2 expression and activity facilitate neuronal plasticity within the brain and contribute to memory retention [59,64]. Thus, our findings, regarding clozapine strongly upregulating COX2 gene expression in human astroglial cells, may be relevant to the beneficial effects of clozapine in the treatment of patients with treatment-resistant SCZ. Over expression of COX2 within the brain, however, has been linked to excitatory neuronal activity, cell injury and inflammation, and has been implicated in SCZ [59]. Although some studies showed elevated levels of COX2 in SCZ, trials using COX2 inhibitors as an adjunct to conventional treatments, did not provide significant therapeutic benefits to patients with SCZ, especially to patients with long-term illness [6,65]. 

The inflammatory response of glial cells is mediated by the phosphatidylinositol-3 (PI3)-Akt signaling pathway, which transmits signals from cytokines, growth factors, hormones or LPS downstream from their cell receptors to transcription factors which modulate gene expression. There is evidence that the phosphorylation of Akt by PI3 leads to activation of NF-kB and to upregulation of gene expression and production of proinflammatory mediators, whereas the inhibition of Akt activities attenuates the inflammatory response [37,66]. 

There are contradictory reports concerning the effect of clozapine on Akt activation. We found that clozapine reduced within 10 min, both the basal and the cytokine-induced Akt phosphorylation, which is in accordance with other reports on clozapine-induced inhibition of Akt activity, followed by attenuation of inflammatory responses [36,37,49,50]. It is possible, therefore, that in our human astroglial cellular model too, the inhibition of Akt phosphorylation by clozapine led to the downregulation of the gene expression and the corresponding production of TNFα, IL-1β and IL-8.

Our results indicate that clozapine upregulates COX2 gene expression through Akt inhibition. This was demonstrated by co-incubation of cytokine mix-stimulated cells with clozapine and the Akt inhibitor MK2206, which resulted in further significant augmentation of COX2 expression, beyond the increase achieved by clozapine alone. Moreover, incubation of cytokine mix-stimulated cells with MK2206 alone significantly increased COX2 expression, implying that inhibition of Akt in human astroglial cells, exposed to cytokine mix, is sufficient for COX2 upregulation. Several studies reported findings similar to ours, namely that inhibition of the PI3-Akt pathway increased COX2 gene expression. Weaver et al. [67] showed that the PI3K inhibitor LY294002 upregulates COX2 mRNA in human colon HT-29 cells. Monick et al. [68] demonstrated in human alveolar macrophages that inhibition of the PI3K pathway augments LPS-induced COX2 mRNA expression and mRNA stability, and De Oliveira et al. [69] found that LY294002 increases COX2 mRNA and protein. 

To the best of our knowledge, this is the first study demonstrating clozapine-induced upregulation of COX2 gene expression in astroglial cells. A recent publication demonstrated a diminished level of COX2 in leukocytes of patients with SCZ; thus, it is possible that upregulation of COX2 achieved by clozapine may be relevant to its efficacy in treatment of chronic and treatment-resistant SCZ. This notion is supported by the evidence that COX2 expression is associated with enhancement of neuronal plasticity, and improvement in cognition and memory. Hence, it seems that clozapine may possess dual beneficial effects: by inhibiting the astroglial production of TNFα, IL-1β and IL-8 it attenuates neuroinflammation while simultaneously improving neuronal brain functions by upregulating COX2. Clozapine-mediated increase in COX2 may, however, account for its detrimental effects, such as hepatotoxicity or cardiotoxicity. Interestingly, chronic administration of clozapine (20 mg/kg/day) to rats exposed to chronic social isolation (21 days), an animal model of depression, induced hepatic oxidative stress, NF-κB activation and increased COX-2 expression [35].

The regulation of COX2 gene expression is a complex process which varies from cell type to cell type and between the same cell types in different species and is stimulus-dependent [70]. Clozapine may exert different effects in different cell types throughout the body, depending on specific pathophysiological conditions. 

### Limitations

The major limitations of this study are the lack of assessment of clozapine on the NF-kB pathway [71]. Previously, clozapine has been shown to downregulate proinflammatory cytokines by targeting NF-κB and Akt phosphorylation [72]. Future studies should, therefore, assess NF-κB phosphorylation levels in the current system as well as in neuronal systems. Additionally, the Akt activity should be conducted using a wider variety of approaches, including knockdown or overexpression of Akt as well as assess Akt phosphorylation dead form in order to elucidate the Akt function in the current context [72].

This study is a pre-clinical and in vitro study; however, its translation to in vivo and bed-side research merits consideration and further investigation. 

Despite these weaknesses, the present study shows the potential of neuro-immunomodulatory activity of clozapine, as a major contributor to the beneficial effect evident in TRS. Future studies should monitor the impact of clozapine treatment on inflammatory mediators in animal models of SCZ and in patients with TRS. In patient studies, the correlation between the anti-inflammatory activity and the antipsychotic activity should be addressed as well. Moreover, such studies should compare the anti-inflammatory activity of clozapine to that of other atypical agents (e.g., olanzapine or risperidone) in order to show the uniqueness of clozapine’s immunomodulatory effect.

## 5. Conclusions

Our study demonstrates that in human astroglial cells, clozapine downregulates the expression and release of the proinflammatory cytokines TNFα, IL-1β and IL-8 while upregulating COX2 expression. Furthermore, clozapine inhibited Akt phosphorylation which may indicate that clozapine inhibition of Akt occurs upstream of COX2 expression. 

These mechanisms may be relevant to clozapine’s unique efficacy in TRS and to its side-effect profile. Further research is required to elucidate the effects of clozapine on COX2 expression and inflammatory mediators in peripheral cells and the relevance to the adverse effects of clozapine. Findings imply that among patients with TRS, those with laboratory evidence of neuroinflammation have the highest potential to benefit from clozapine treatment. 

While this is an in vitro study, the findings suggest that the anti-inflammatory properties of clozapine may be involved in its mechanism of action and such activity may be pertinent to its unique efficacy in TRS. The clozapine-induced suppressive effect on the immune system may be of benefit to the inflamed brain but its impact on the peripheral immune system is unclear. Since bipolar disorder, major depressive disorder and autism spectrum disorder are also claimed to be associated with neuroinflammation, clozapine may be beneficial for those disorders too when they prove drug-resistant [73].

## Figures and Tables

**Table 1:** The immunomodulatory impact of clozapine on the astroglial cells—summary of major results.

Inflammatory Mediator	Relative mRNA Expression				Relative Protein Production			
	Cytokine Mix	Cytokine Mix + Clozapine	t	*p*	Cytokine Mix	Cytokine Mix + Clozapine	t	*p*
	%	% ±SEM			%	% ±SEM		
TNFα	100	63.4 ± 6.5	5.68	<0.001	100	81 ± 4	4.07	<0.05
IL-1β	100	63.6 ± 5.2	4.78	<0.001	100	73 ± 1.4	8.48	<0.001
IL-8	100	76.1 ± 3.6	13.46	<0.0001	100	35 ± 4.1	8.51	<0.001
COX2	100	186 ± 7.5	11.67	<0.0001				

## Data Availability

Data are available on request from the corresponding author.
